# Release of glucose repression on xylose utilization in *Kluyveromyces marxianus* to enhance glucose-xylose co-utilization and xylitol production from corncob hydrolysate

**DOI:** 10.1186/s12934-019-1068-2

**Published:** 2019-02-01

**Authors:** Yan Hua, Jichao Wang, Yelin Zhu, Biao Zhang, Xin Kong, Wenjie Li, Dongmei Wang, Jiong Hong

**Affiliations:** 1 School of Life Sciences, University of Science and Technology of China, Hefei, 230027 Anhui People’s Republic of China; 2 Hefei National Laboratory for Physical Science at the Microscale, Hefei, 230026 Anhui People’s Republic of China; 3 CAS Key Lab of Biobased Materials, Qingdao Institute of Bioenergy and Bioprocess Technology, Chinese Academy of Sciences, Qingdao, 266101 China

**Keywords:** *Kluyveromyces marxianus*, Glucose repression, *Km*HXK1, *Km*MIG1, Glucose and xylose co-utilization, Corncob hydrolysate, Xylose mother liquor, Inexpensive organic nitrogen source

## Abstract

**Background:**

Lignocellulosic biomass is one of the most abundant materials for biochemicals production. However, efficient co-utilization of glucose and xylose from the lignocellulosic biomass is a challenge due to the glucose repression in microorganisms. *Kluyveromyces marxianus* is a thermotolerant and efficient xylose-utilizing yeast. To realize the glucose–xylose co-utilization, analyzing the glucose repression of xylose utilization in *K. marxianus* is necessary. In addition, a glucose–xylose co-utilization platform strain will facilitate the construction of lignocellulosic biomass-utilizing strains.

**Results:**

Through gene disruption, hexokinase 1 (*Km*HXK1) and sucrose non-fermenting 1 (*Km*SNF1) were proved to be involved in the glucose repression of xylose utilization while disruption of the downstream genes of cyclic AMP-protein kinase A (cAMP-PKA) signaling pathway or sucrose non-fermenting 3 (SNF3) glucose-sensing pathway did not alleviate the repression. Furthermore, disruption of the gene of multicopy inhibitor of GAL gene expression (*Km*MIG1) alleviated the glucose repression on some nonglucose sugars (galactose, sucrose, and raffinose) but still kept glucose repression of xylose utilization. Real-time PCR analysis of the xylose utilization related genes transcription confirmed these results, and besides, revealed that xylitol dehydrogenase gene (*KmXYL2*) was the critical gene for xylose utilization and stringently regulated by glucose repression. Many other genes of candidate targets interacting with SNF1 were also evaluated by disruption, but none proved to be the key regulator in the pathway of the glucose repression on xylose utilization. Therefore, there may exist other signaling pathway(s) for glucose repression on xylose consumption. Based on these results, a thermotolerant xylose–glucose co-consumption platform strain of *K. marxianus* was constructed. Then, exogenous xylose reductase and xylose-specific transporter genes were overexpressed in the platform strain to obtain YHY013. The YHY013 could efficiently co-utilized the glucose and xylose from corncob hydrolysate or xylose mother liquor for xylitol production (> 100 g/L) even with inexpensive organic nitrogen sources.

**Conclusions:**

The analysis of the glucose repression in *K. marxianus* laid the foundation for construction of the glucose–xylose co-utilizing platform strain. The efficient xylitol production strain further verified the potential of the platform strain in exploitation of lignocellulosic biomass.

**Electronic supplementary material:**

The online version of this article (10.1186/s12934-019-1068-2) contains supplementary material, which is available to authorized users.

## Background

Lignocellulosic biomass, composed of cellulose, hemicellulose, and lignin, is an important renewable and abundant resource. With certain pretreatment, various sugars (mainly glucose and xylose) can be released from the biomass and used for manufacture of value-added products [1, 2]. However, xylose, as the second abundant sugar forming hemicellulose, is difficult to be effectively co-utilized by industrial microorganisms due to the glucose repression [3]. Therefore, to improve the economic benefits of lignocellulosic biomass, efficient utilization of xylose, or co-utilization of glucose and xylose is necessary.

Many efforts have been made to create “optimized” strains that can effectively and simultaneously utilize the xylose and glucose in lignocellulosic biomass [3, 4]. However, for certain chemicals production, correspondent modifications are strain-dependent [5, 6]. Therefore, a xylose–glucose co-consumption platform strain would largely facilitate the construction of desired strains and the exploitation of lignocellulosic biomass. *Kluyveromyces marxianus* is known as a “generally regarded as safe” (GRAS) microorganism and able to assimilate various sugars including xylose [7]. It is also famous for its high growth rate at an elevated temperature, which means reduced cooling cost, increased fermentation rate, and minimized risk of contamination in industrial fermentation [8, 9]. Therefore, *K. marxianus* is a good candidate for industrial utilization of lignocellulosic biomass. Though xylose can be utilized efficiently by engineered *K. marxianus*, the utilization is repressed by glucose [10, 11]. As a result, construction of a xylose–glucose co-consumption platform is preferred to biochemicals production from lignocellulosic biomass. Nonetheless, few studies have been performed to unravel the glucose repression in *K. marxianus*. There are many works to clarify the glucose repression in *Saccharomyces cerevisiae* [4], however, the reports on glucose repression of the endogenous xylose utilization are few due to poor native xylose assimilation capability of *S. cerevisiae* [12]. Even with less genes in genome (4912 open reading frames for NBRC1777) than *S. cerevisiae* [13], *K. marxianus* is an efficient xylose-utilizing yeast which is different to *S. cerevisiae* and it is not surprising to find novel glucose repression mechanism in *K. marxianus* [14]. Therefore, the analysis of the glucose repression is necessary before construction of the xylose–glucose co-utilization platform strain of *K. marxianus.*

In this study, the glucose repression pathway in *K. marxianus* was analyzed through a series of genes disruption, and a xylose–glucose co-consumption platform strain was constructed. Finally, based on the platform, we constructed a strain that efficiently utilized the glucose and xylose from corncob hydrolysate or xylose mother liquor for xylitol production.

## Methods and materials

### Reagents and microorganisms

Chemicals used here were all of analytical grade or higher. d-glucose, d-xylose, xylitol, arabinose, arabitol, 2-deoxy-d-glucose (2-DG), and yeast nitrogen base without amino acids (YNB) were purchased from Sangon Biotech Co. (Shanghai, China), whereas yeast extract (YE; LP0021), tryptone (LP0042), and peptone (LP0037) were from Oxoid (Oxoid Ltd., Basingstoke, Hampshire, England). Besides, YE (FM902) and peptone (FP320) were obtained from Angel (Angel Yeast Co., Ltd, China). Corn steep liquor (CSL) was acquired from Fangqi Co. (Shanghai, China), and defatted soybean meal (DSM) was from Enzyme Co. (Shandong, China). Xylose mother liquor (XML) was obtained from Longlive Bio-technology Co., Ltd. (Shandong, China). Restriction endonuclease and T4 DNA ligase were bought from Thermo Fisher Scientific (West Palm Beach, Florida, USA), whereas *K. marxianus* NBRC1777 was obtained from NITE Biological Resource Center (Tokyo, Japan). *K. marxianus* YHJ010 is a *TRP1*, *LEU2*, and *URA3* auxotrophic strain derived from NBRC1777 [15]. The YE–peptone–dextrose/glycerol (YPD/YPG) medium (10 g/L Oxoid YE, 20 g/L Oxoid peptone, 20 g/L glucose, or 20 g/L glycerol) was used for cultivation of *K. marxianus*. The synthetic dropout (SD) medium (6.7 g/L YNB and 20 g/L glucose) was used to screen transformants with appropriate supplements. *Escherichia coli* DH5α was served as the host for gene cloning and was grown in lysogeny broth (LB) medium (5 g/L Oxoid YE, 10 g/L tryptone, 10 g/L NaCl). For solid plates, 15 g/L agar was added to each medium.

### Construction of plasmids and strains

The plasmids and primers involved are described in Additional file 1: Tables S1 and S2, respectively. Genes coding for adenylate cyclase (*KmCYR1*), GTPaes (*KmRAS*), sucrose non-fermenting (*KmSNF1*), multi-copy inhibitor of GAL gene expression (*KmMIG1*), CATabolite repression (*KmCAT8*), alcohol dehydrogenase II synthesis regulator (*KmADR1*), negative regulator of glucose-repressed genes (*KmNRG1*), multicopy suppressor of SNF1 mutation (*KmMSN2*), regulator of drug sensitivity (*KmRDS2*), and restores glucose transport (*KmRGT1*) were amplified from the genomic DNA of *K. marxianus* YHJ010 with primers of KmCYR1H2F and KmCYR1H2R, KmRASHF and KmRASHR, KmSNF1HF and KmSNF1HR, KmMIG1F and KmMIG1R, KmCAT8HF and KmCAT8HR, KmADR1HF and KmADR1HR, KmNRG1HF and KmNRG1HR, KmMSN2F and KmMSN2R, KmRDS2F and KmRDS2R, and KmRGT1F and KmRGT1R (Additional file 1: Table S2), respectively, and their GenBank accession numbers are listed in Table 1. The obtained DNA fragments were inserted into pGEM-T Easy (Promega, Madison, WI, USA) to obtain plasmids pKmCYR1, pKmRAS, pKmSNF1, pKmMIG1, pKmCAT8, pKmADR1, pKmNRG1, pKmMSN2, pKmRDS2, and pKmRGT1 (Additional file 1: Table S1). Then the plasmids containing disruption cassettes of the various genes were constructed as follows. The expression cassette of *ScURA3* was amplified from the plasmid yEUGAP with primers SCURA3-SMAI-FULL-F and SCURA3-SMAI-FULL-R (Additional file 1: Table S2) and digested with *Sma*I. The pKmCYR1, pKmRAS, pKmSNF1, and pKmADR1 plasmids were digested with *Eco*RV to remove part of the ORF sequence and dephosphorylated with FastAP (Thermo Fisher Scientific, West Palm Beach, FL, USA). The *ScURA3* was ligated with pKmCYR1, pKmRAS, pKmSNF1, and pKmADR1 fragment to obtain pKmCYR1-U, pKmRAS-U, pKmSNF1-U, and pKmADR1-U, respectively (Additional file 1: Table S1). The frame of plasmid and part sequence of *KmCAT8*, *KmNRG1*, *KmMIG1*, *KmMSN2*, *KmRDS2*, or *KmRGT1* was amplified using above correspondent plasmid as template with primers of dKmCAT8F and dKmCAT8R, dKmNRG1F and dKmNRG1R, dKmMIG1F and dKmMIG1R, dKmMSN2F and dKmMSN2R, dKmRSD2F and dKmRSD2R, or dKmRGT1F and dKmRGT1R, respectively (Additional file 1: Table S2). Then each amplified fragment was ligated with the *ScURA3* to obtain plasmids pKmCAT8-U, pKmNRG1-U, pKmMIG1-U, pKmMSN2-U, pKmRDS2-U, and pKmRGT1-U (Additional file 1: Table S1). In these plasmids, each gene DNA with ORF partly substituted by *ScURA3* was used as the disruption cassette.

**Table 1:** The GenBank accession numbers of the genes used in this study

Gene name	GenBank accession no.
*KmCYR1*	BAP71016.1
*KmRAS*	BAP73921.1
*KmSNF1*	BAP71360.1
*KmMIG1*	BAP70066.1
*KmCAT8*	BAP73751.1
*KmADR1*	BAP70124.1
*KmNRG1*	BAP71632.1
*KmMSN2*	BAP71827.1
*KmRDS2*	BAP70766.1
*KmRGT1*	BAP73047.1

The yeast strains employed in this study are summarized in Table 2. After the gene disruption cassettes were amplified from pKmCYR1-U, pKmRAS-U, pKmSNF1-U, pKmMIG1-U, pKmCAT8-U, pKmADR1-U, pKmNRG1-U, pKmMSN2-U, pKmRDS2-U, and pKmRGT1-U, they were transformed into strain YHJ010 by the lithium acetate method [16], respectively, to obtain knockout strains YΔCYR1, YΔRAS, YΔSNF1, YΔMIG1, YΔCAT8, YΔADR1, YΔNRG1, YΔMSN2, YΔRDS2, and YΔRGT1 (Table 2). The plasmids for heterologous genes expression (Additional file 1: Table S1) were linearized by *Sma*I digestion and then transformed into *K. marxianus*. Strains YLM001 and YLM002 with hexokinase (*KmHXK1*) or glucokinase *(KmGLK1)* gene disruption were constructed in our previous study which were used for enzymatic identification and characterization [17]. Strain YLM005 was constructed via overexpression of *KmGLK1* in YLM001 [17]. The *ScURA3* gene in *K. marxianus* YLM005 was disrupted to recover the URA3 selection marker by using *ScURA3* disruption cassette, which was amplified from pMD18T-ΔScURA3 [11]. The disrupted strain was selected on an SD plate containing uracil and 0.1% 5′-fluoroorotic acid (5′-FOA) and was named YHY003 (Table 2). Linearized plasmid pZJ011 [11], which contains two *Neurospora crassa* (*NcXYL1*) expression cassettes under the constitutive promoters of *TDH3* from *S. cerevisiae* and *K. marxianus*, respectively, was transformed into strain YHY003 to obtain YHY006 (Table 2). Then, the plasmid pZJ012 [11] was introduced into YHY006 to create YHY008 (Table 2), which overexpressed another two copies of *NcXYL1*. The *ScURA3* gene in YHY008 was disrupted again to recover the selection marker and strain YHY009 was obtained (Table 2). Linearized plasmid pZJ061 was transformed into YHY009 to obtain strain YHY010 (Table 2), in which *ScGal2N376F* gene (xylose-specific transporter and moderately insensitive to glucose competition) [18] was expressed to release glucose repression at the transport stage. The *ScURA3* gene in YHY010 was disrupted and strain YHY011 was obtained (Table 2). Then, plasmid pZJ061 was introduced into YHY011 to obtain strain YHY013, which expresses two copies of the xylose-specific transporter *ScGAL2N376F* (Table 2).

**Table 2:** *K. marxianus* strains used in this study

Strains	Description	References
YHJ010	NBRC1777, Δ*KmURA3*::*KAN*^*R*^, Δ*KmLEU2*::*HISG*, Δ*KmTRP1*::*HISG*	[15]
YLM001	YHJ010, Δ*KmHXK1::ScURA3*	[17]
YLM002	YHJ010, Δ*KmGLK1::ScURA3*	[17]
YLM005	YLM001, YEGAP-*KmGLK1*	[17]
YWD016	YHJ010, *ScURA3*	[17]
YΔSNF1	YHJ010, Δ*KmSNF1*::*ScURA3*	This study
YΔCYR1	YHJ010, Δ*KmCYR1*::*ScURA3*	This study
YΔRAS	YHJ010, Δ*KmRAS*::*ScURA3*	This study
YΔMIG1	YHJ010, Δ*KmMIG1*::*ScURA3*	This study
YΔADR1	YHJ010, Δ*KmADR1*::*ScURA3*	This study
YΔCAT8	YHJ010, Δ*KmCAT8*::*ScURA3*	This study
YΔMSN2	YHJ010, Δ*KmMSN2*::*ScURA3*	This study
YΔNRG1	YHJ010, Δ*KmNRG1*::*ScURA3*	This study
YΔRDS2	YHJ010, Δ*KmRDS2*::*ScURA3*	This study
YΔRGT1	YHJ010, Δ*KmRGT1*::*ScURA3*	This study
YHY003	YLM005, Δ*ScURA3*	This study
YHY006	YHY003, pZJ011, 2 copies of *NcXYL1*	This study
YHY008	YHY006, pZJ012, 4 copies of *NcXYL1*	This study
YHY009	YHY008, Δ*ScURA3*	This study
YHY010	YHY009, pZJ061, 1 copy of *ScGAL2N376F*	This study
YHY011	YHY010, Δ*ScURA3*	This study
YHY013	YHY011, pZJ061, 2 copies of *ScGAL2N376F*	This study

### Evaluation of glucose repression in *K. marxianus* with gene disrupted

The glucose analogue 2-deoxyglucose (2-DG) can be taken up into cells by hexose transporters, phosphorylated by hexokinase and enable the cells produce glucose repression signal, but cannot be metabolized for growth. As a result, 2-DG resistibility can be used for evaluating glucose repression of yeast [19, 20]. Here, strain YWD016 (YHJ010 + *ScURA3*) (Table 2) was used as non-disruption control. Firstly, all the strains except YΔSNF1 were precultured in 5 mL of YPG, while YΔSNF1 was precultured in YPD medium for its growth defect on YPG. After overnight cultivation at 37 °C and with 250 rpm shaking, the cells were collected by centrifugation at 5000×*g* for 5 min and resuspended with sterilized water. The diluted cell suspensions were spotted on YP plates containing various carbon sources (glucose, xylose, galactose, sucrose, or raffinose) with or without 0.01% 2-DG and then incubated at 42 °C or 30 °C.

### Real-time PCR analysis

The relative expression levels of xylose reductase gene (*KmXYL1*), xylitol dehydrogenase gene (*KmXYL2*), and xylulokinase gene (*KmXYL3*) in strain YWD016, YLM001, YLM002, YΔMIG1, and YLM005 were determined using real-time PCR (RT-PCR). The primers used are shown in Additional file 1: Table S2. Because the concentrations of glucose and xylose in XML are ~ 10% and 40%, and the concentration of glucose in corncob hydrolysate is even lower, a mixture containing 20 g/L glucose and 80 g/L xylose was employed. After cultivated in 5 mL of YPG medium at 37 °C overnight, YWD016, YΔMIG1, and YLM005 were transferred into 250 mL Erlenmeyer flasks containing 30 mL of YPD (20 g/L glucose), YPDX (20 g/L glucose and 80 g/L xylose), or YPX (80 g/L xylose) medium at an initial optical density at 600 nm (OD_600_) of 1.0 (0.42 g of dried cells per liter), and then shaken at 250 rpm and 42 °C. As the OD_600_ reached 4.0, total RNA was isolated using yeast total RNA extraction kit (Sangon Biotech Co. Shanghai, China). The cDNA was synthesized by ReverTra Ace qPCR RT Master Mix kit (Toyobo, Japan) and real-time PCR was conducted on a Step One Plus Real-Time PCR system (Thermo Fisher Scientific, West Palm Beach, FL) with AceQ qPCR SYBR Green Master Mix kit (Vazyme biotech co., China). Gene *KmACT1* was used as an internal control.

### Evaluation of the glucose–xylose co-fermentation ability of the platform strain and the strains with further genetic modifications

The glucose–xylose co-consuming and xylitol producing ability of *K. marxianus* YLM005 (Table 2) and its derivative strains, including YHY006, YHY008, YHY010, and YHY013 (Table 2), were evaluated in medium containing xylose and glucose. After cultivated in 5 mL of the YPD medium overnight at 37 °C, the cells were transferred into 250 mL Erlenmeyer flasks containing 30 mL of the YPDX medium (20 g/L glucose and 80 g/L xylose) with an initial OD_600_ of 1.0, and then cultivated at 42 °C with 250 rpm shaking. The glucose and xylose consumption and xylitol production during the fermentation were determined by high performance liquid chromatography (HPLC).

### Evaluation of xylitol production from glucose–xylose mixture with various nitrogen sources by *K. marxianus* YHY013

After strain YHY013 was proved to be the most efficient xylitol-producing strain, various nitrogen sources (YE, peptone, and cheap organic nitrogen source including CSL and DSM) were evaluated for xylitol production by YHY013. Each kind of nitrogen source was supplied at a final concentration of 20 g/L in single nitrogen source experiments. The total concentration of nitrogen source was also kept at 20 g/L during compounding nitrogen source evaluation including 10 g/L each nitrogen source. The carbon source for evaluation was 20 g/L glucose and 80 g/L xylose or 30 g/L glucose and 120 g/L xylose.

### Xylitol production in a fermenter

After the effect of nitrogen sources was evaluated, a batch fermentation was conducted in a benchtop fermenter (Bioflo 110, New Brunswick Scientific, Edison, New Jersey, USA) in duplicate. The seed culture was incubated in a 250 mL Erlenmeyer flask containing YPD medium at 37 °C with agitation at 250 rpm. After the cells were collected, they were inoculated into the fermenter containing the fermentation medium (10 g/L CSL, 10 g/L DSM, 30 g/L glucose and 120 g/L xylose, or 50 g/L glucose and 200 g/L xylose, or diluted XML, or the concentrated corncob hydrolysate) at initial OD_600_ = 1. The fermentation temperature was maintained at 42 °C, while agitation speed and oxygen flux were optimized for xylitol production.

### Preparation of corncob hemicellulose hydrolysate

The corncob hydrolysate was prepared as previously reported [5] with some modifications. The diluted acid [0.5% (w/w) H_2_SO_4_ + 1.5% (w/w) H_3_PO_4_)] was employed to hydrolyze the corncob with a solid: liquid (w/v) ratio of 1:4. The nondetoxified hydrolysate was obtained by adding lime cream into the pretreated corncob solution until pH reached 6.0. The detoxified hydrolysate was prepared through over-lime and adsorption by 2% (w/w) activated charcoal powder (Sangon, Shanghai). The detoxified or nondetoxified hydrolysate was recovered by vacuum filtration and concentrated in vacuum at 70 °C. Both the nondetoxified and detoxified hydrolysate were used directly (without sterilization) for fermentation.

### Analytical methods

High performance liquid chromatograph (HPLC) with a ROA-Organic Acid H+ (8%) column (Phenomenex, USA) was conducted to quantify d-glucose, D-xylose, xylitol, arabinose, and arabitol; 0.0025 M H_2_SO_4_ was served as the mobile phase at a column temperature of 75 °C and a flow rate of 0.3 mL/min. The concentrations of furfural and 5-hydroxymethylfurfural (5-HMF) were analyzed using a C18 column (Phenomenex, USA) at a column temperature of 30 °C and a flow rate of 0.3 mL/min. The mobile phase was a mixture of water and methanol (80:20, v/v) with detection at a wavelength of 285 nm. The culture was centrifuged at 14,000×*g* for 10 min, and the supernatant was diluted 20-fold for HPLC analysis.

## Results and discussion

### The disruption of *KmHXK1* released the glucose repression of xylose utilization

**Fig. 1:**
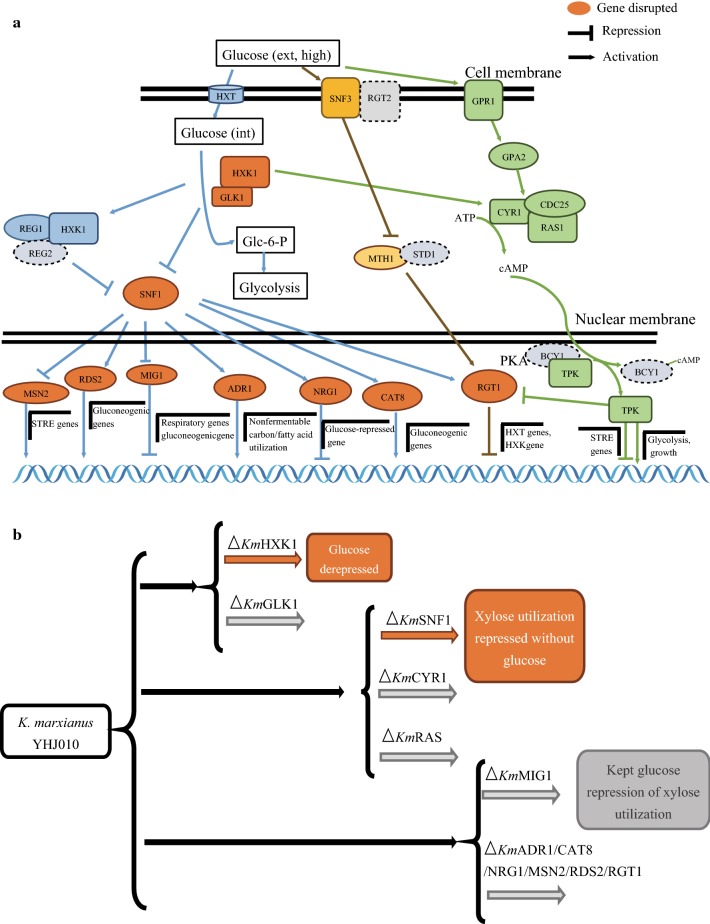
The putative pathway and analysis schematic diagram of glucose repression in *K. marxianus.* **a** Putative signaling pathway of glucose repression in *K. marxianus*. The orange color icons indicated that the correspondent gene was disrupted and the dash icons indicated that the gene was not found in genome of *K. marxianus* based on theoretical translation or homology analysis; the circles indicate regulators, the squares indicate glucose sensors, while rectangles indicate enzymes. **b** Schematic diagram of the analysis process on glucose repression of xylose utilization in *K. marxianus*. HXK1: hexokinase; GLK1: glucokinase; SNF1: sucrose non-fermenting, AMP-activated S/T protein kinase; CYR1: adenylate cyclase; RAS: GTPaes; MIG1: multicopy inhibitor of GAL gene expression; ADR1: alcohol dehydrogenase ii synthesis regulator; CAT8: CATabolite repression; NRG1: negative regulator of glucose-repressed genes; MSN2: multicopy suppressor of SNF1 mutation; RDS2: regulator of drug sensitivity; RGT1: restores glucose transport

**Fig. 2:**
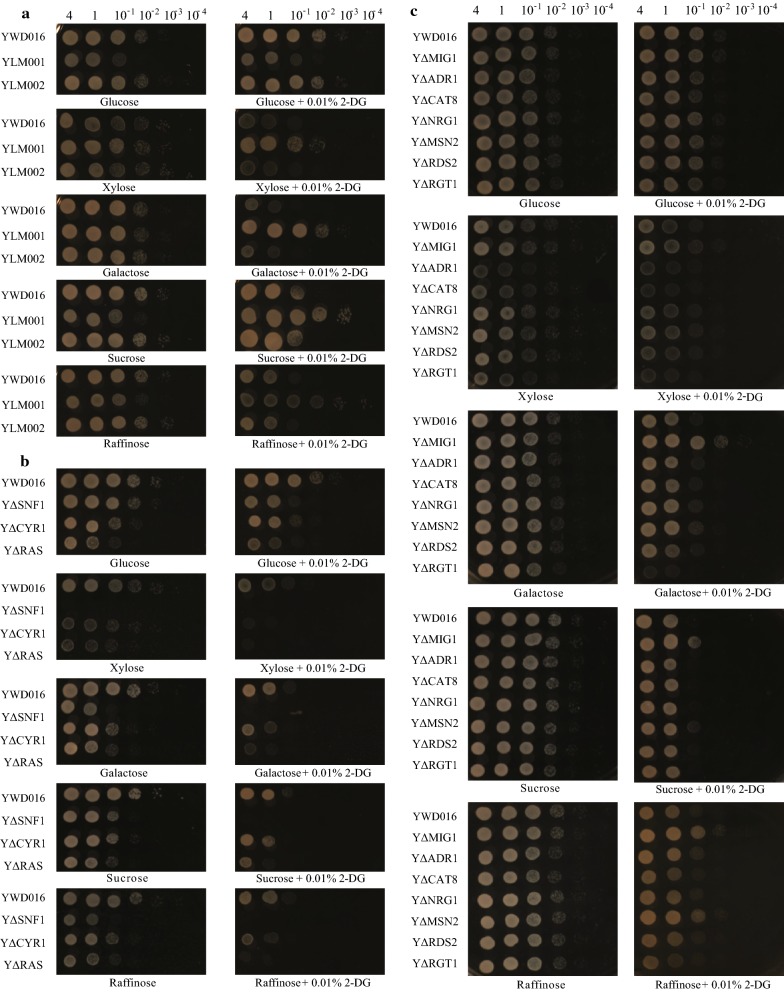
The growth of strains on YP plates containing various sugars with or without 0.01% 2-DG at 42 °C. **a** YWD016, YLM001 (Δ*KmHXK1*) and YLM002 (Δ*KmGLK1*), **b** YWD016, YΔSNF1, YΔCYR1, and YΔRAS, and **c** YWD016, YΔMIG1, YΔADR1, YΔCAT8, YΔNRG1, YΔMSN2, YΔRDS2, and YΔRGT1. YWD016 is a URA3 complement strain of YHJ010 (Table 2) and served as a non-disruption control. (Left to right, OD_600_ = 4, 1, 10^−1^, 10^−2^, 10^−3^, 10^−4^)

There are two hexokinases (HXK1 and 2) and one glucokinase (GLK) reported in *S. cerevisiae* and the glucose phosphorylation initiated by HXK2 is crucial for glucose repression [21]. HXK2 generates signals and results in transcriptional repression of the genes involved in nonglucose sugars assimilation (Fig. 1a) [21, 22]. However, only one HXK and one GLK were identified in *K. marxianus* in our previous study [17]. For this reason, *KmHXK1* and *KmGLK1* were selected for investigation. As the results showed, strain YLM001 (Δ*KmHXK1*) grew well on YP plates containing 2% xylose or other nonglucose sugars (galactose, sucrose, or raffinose) with 0.01% 2-DG, while the YLM002 (Δ*KmGLK1*) and control strain YWD016 can hardly grow (Fig. 2a). It indicated that *Km*HXK1, the only hexokinase in *K. marxianus* [17], can generate glucose repression-related signals and its disruption released the utilization of xylose and other nonglucose sugars from glucose repression. On the other hand, the disruption of *KmGLK1* which is nonessential for hexose phosphorylation in *K. marxianus* [17] did not cause any phenotypic change.

### *Km*SNF1 is also responsible for the glucose repression of xylose utilization

In *S. cerevisiae*, the glucose phosphorylation signal is generated from HXK, then it is transmitted to the main glucose repression pathway (SNF1/MIG1) or linked to the RAS/protein kinase A (PKA) signaling pathway and both of them are involved in nonglucose sugars metabolism [23] (Fig. 1a). Therefore, the homologous genes correspondent to SNF1, RAS or CYR1 which play a vital role in these pathways of *S. cerevisiae* were disrupted in YHJ010 to figure out whether these pathways are also involved in glucose repression of *K. marxianus*. As shown in Fig. 2b, the growth of strain YΔSNF1 with xylose was completely repressed even without 2-DG while the growth with glucose was not affected. In addition, the growth of YΔSNF1 with other nonglucose sugars was weaker than that with glucose (Fig. 2b). It is well known that the activity of *S*cSNF1 is repressed with the presence of glucose and its activity is indispensable for many nonglucose sugars metabolism [23, 24]. The growth defect of YΔSNF1 on YPX indicated that the utilization of xylose in *K. marxianus* also mainly relies on the activity of SNF1. On the other hand, blocking the PKA pathway (disruption of RAS or CYR1) of *K. marxianus* only caused weaker growth even with glucose (Fig. 2b) which was consistent with that of *S. cerevisiae* [3, 25]. Whereas, the disruption of CYR1 or RAS did not release glucose repression on plates with 2-DG. Therefore, *Km*SNF1 pathway was the main signaling pathway for the glucose repression of xylose utilization in *K. marxianus*.

### The main signaling pathway of glucose repression on xylose utilization is not via *KmMIG1* in *K. marxianus*

MIG1 is the target of SNF1 and its deletion alters the sugar preference and metabolic patterns of *S. cerevisiae* [26, 27]. Besides, *Km*MIG1 was proved to be fully functional when expressed in *S. cerevisiae* [28]. The disruption of *KmMIG1* was expected to release the utilization of xylose from glucose repression in *K. marxianus*. Nevertheless, the disruption of *KmMIG1* only released glucose repression of galactose, sucrose, and raffinose utilization, while the growth on xylose was still repressed with 2-DG (Fig. 2c). These results indicated that the main signaling pathway for glucose repression on xylose utilization was not via MIG1 in *K. marxianus.* Then many other candidate target proteins of *Km*SNF1 as shown in Fig. 1a were disrupted in YHJ010 to explore other potential pathway(s) for glucose repression of xylose utilization. In *S. cerevisiae*, ADR1, CAT8 and RDS2 are involved in the nonfermentive carbon utilization or gluconeogenic gene expression regulation [29]; the NRG1 is another regulator that inactivates multiple glucose-repressed genes expression (like *GAL, SUC2* gene) [30]. However, disruption of *KmADR1*, *KmCAT8*, *KmRDS2*, or *KmNRG1* in *K. marxianus* (Fig. 1a), which were found in genome based on theoretical translation and homology analysis, did not change the glucose repression of xylose metabolism (Fig. 2c). In *S. cerevisiae*, RGT1 is the target of SNF3/RGT2 glucose-sensing pathway that regulated by glucose-induced degradation of MTH1 and STD1 [31], and it is also regulated by SNF1 and PKA through phosphorylation in the glucose repression signaling pathway [32, 33] (Fig. 1a). Thus, *KmRGT1* was disrupted to evaluate the effect on glucose repression. However, the strain YΔRGT1 was able to grow on YPX and the growth was still inhibited with 2-DG (Fig. 2c). Therefore, disruption of *KmRGT1* did not alter the glucose repression. A recent report suggests that stress response has a relationship with the alleviation of glucose repression [34]. Thus, the genes of proteins correspondent to the regulators in stress response (MSN2 and MSN4) in *S. cerevisiae* were also disrupted. After the *KmMSN2*, the sole *MSN* in *K. marxianus*, was disrupted, the glucose repression on xylose utilization was still repressed (Fig. 2c). Besides, when cultured at 30 °C, the glucose repression of disruption strains was the same as that with culture temperature of 42 °C (Additional file 2: Fig. S1). It indicated that the phenotypes in Fig. 2 were not evoked by the elevated temperature. Therefore, *KmHXK1* and *KmSNF1* were the key genes involved in the glucose repression pathway of *K. marxianus*. *Km*MIG1 was involved in the glucose repression on nonglucose sugars like galactose in *K. marxianus*, which is consistent with previous study [35]. Nonetheless, strain with *Km*MIG1 disruption did not release the utilization of xylose from glucose repression. Although the target gene of *Km*SNF1 for glucose repression on xylose utilization was still unknown, the results here laid the foundation for further clarification of the repression mechanism of *K. marxianus* and contributed to the construction of a glucose repression released platform strain.

### A platform strain with glucose repression released

**Fig. 3:**
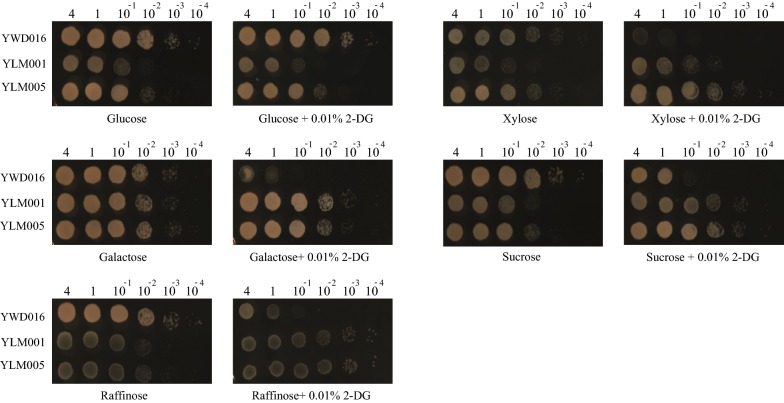
The growth of strains on YP plates containing different sugars with or without 0.01% 2-DG. YLM001 is the strain with *Km*HXK disruption and YLM005 is the strain with overexpression of *Km*GLK in YLM001. YWD016 is a URA3 complement strain of YHJ010 (Table 2) and served as a non-disruption control. (left to right, OD_600_ = 4, 1, 10^−1^, 10^−2^, 10^−3^, 10^−4^)

The disruption of HXK1 released glucose repression of YLM001 (Δ*KmHXK1*) while led a severe defect in glucose metabolism, which is consistent with that in *S. cerevisiae* and limits its further application [36]. In previous study about enzymatic characterization, overexpression of *KmGLK1* in YLM001 could recover its glucose assimilation ability and reverse the growth defect on glucose [17], but whether it still remains the release of glucose repression is unknown. Here, the glucose repression in YLM005 (*Km*GLK1 overexpressed in YLM001) was evaluated on YP plate with nonglucose sugars and 0.01% 2-DG, and the results showed that YLM005 kept glucose derepression of xylose and other nonglucose sugars utilization (Fig. 3). The results were consistent with the real time PCR results in following section. Therefore, *K. marxianus* YLM005 can be used as a platform strain for co-consumption of xylose and glucose.

### *KmXYL2* is the key target gene for glucose repression of xylose utilization

In *K. marxianus*, xylose reductase, xylitol dehydrogenase, and xylulokinase convert the xylose into xylulose-5-P, and then xylulose-5-P enters the pentose phosphate pathway. In order to check key step of the glucose repression on xylose utilization in *K. marxianus*, the expression levels of the xylose metabolism related genes (*KmXYL1*, *KmXYL2*, and *KmXYL3*) were analyzed by quantitative RT-PCR.

**Fig. 4:**
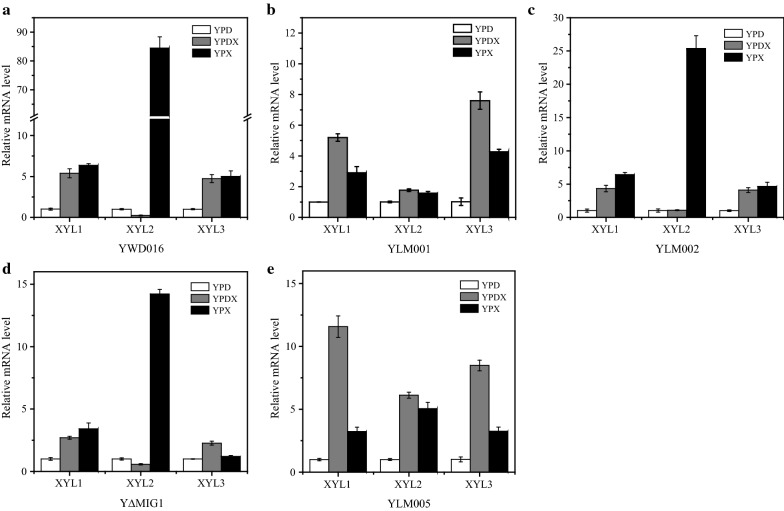
Relative expression of *KmXYL1*, *KmXYL2*, and *KmXYL3* of strains cultivated with YPD, YPDX, or YPX. **a** YWD016, **b** YLM001, **c** YLM002, **d** YΔMIG1, and **e** YLM005. All values are plotted with reference to strains grown in YPD medium and are the means of three biological replicates ± standard deviation

The expression level of *KmXYL2* of YWD016 with YPX was significantly higher than that with YPD, with a fold change of 84.51, while the fold changes of *KmXYL1* and *KmXYL3* were 6.37 and 5.0, respectively (Fig. 4a). With YPDX (20 g/L glucose and 80 g/L xylose), however, *KmXYL2* in YWD016 was hardly expressed, whereas the expression levels of *KmXYL1* and *KmXYL3* were still increased with fold changes of 5.39 and 4.73, respectively (Fig. 4a). The relative expression levels of these three genes in YLM002 were similar to that in YWD016 (Fig. 4c). The results indicated that *KmXYL2* was the key target gene for the glucose repression of xylose utilization. Contrary to the expression of *KmXYL2*, the transcription of *KmXYL1* and *KmXYL3* was not repressed by glucose, and still induced when cultured with YPDX.

In YΔMIG1, the expression of *KmXYL1* and *KmXYL3* was inducible by xylose even with glucose while the *KmXYL2* expression was still repressed by glucose (Fig. 4d). When cultivated in YPX, the expression of *KmXYL1*, *KmXYL2*, and *KmXYL3* increased with fold changes of 3.42, 14.22, 1.27, respectively (the fold changes of expression in YPD were all set as 1). However, when cultivated in YPDX, the expression of *KmXYL2* was repressed with a fold change of 0.57, and the *KmXYL1* and *KmXYL3* increased with fold changes of 2.70 and 2.27, respectively (Fig. 4d). Therefore, the glucose repression in YΔMIG1 was not released.

Contrary to YΔMIG1 and YWD016, the expression of *KmXYL2* in YLM001 and YLM005 cultivated with YPDX was induced with a fold change even higher than that with YPX (Fig. 4b, e), indicating that the glucose repression on xylose utilization of YLM001 and YLM005 was released. It is interesting to note that the fold changes of *KmXYL1* and *KmXYL3* in YLM001 and YLM005 cultivated with YPDX were also higher than that with YPX. It is possible that cells grow better with glucose than with xylose [37] and further enhanced these genes transcription by xylose induction with glucose repression released.

In conclusion, *KmXYL2* is the key target gene for glucose repression of xylose utilization. The GLK1 and MIG1 disruption did not release glucose repression on YPX plate with 2-DG (Fig. 2c) and the expression of *KmXYL2* was stringently repressed when cultured with YPDX (Fig. 4c, d). These results indicated that GLK1 and MIG1 were not critical for glucose repression of xylose utilization. On the other hand, the obvious growth advantage on YPX plate with 2-DG (Fig. 3) and the relative high transcription levels of *KmXYL1*, *KmXYL2*, and *KmXYL3* (Fig. 4e) with YPDX medium enable YLM005 as a glucose derepression platform strain. The advantages of this platform were evaluated in the later work for constructing an efficient glucose–xylose co-utilization strain.

### Improved xylitol production from glucose and xylose mixture by *K. marxianus* YLM005 and the strains with further genetic modifications

**Fig. 5:**
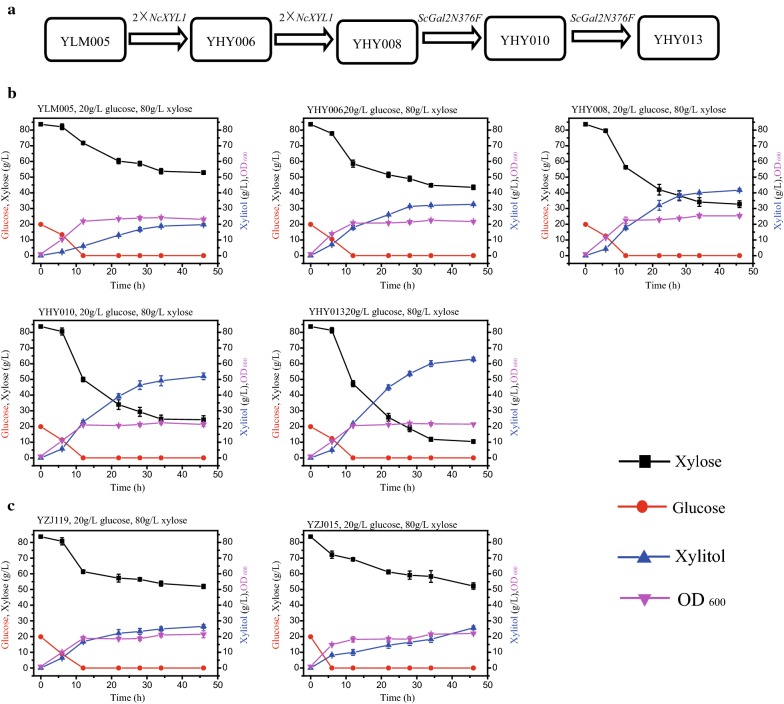
Improved xylitol production from glucose and xylose mixture by *K. marxianus* YLM005 and its derivatives. **a** A schematic diagram showing construction of the strain derived from YLM005. The co-fermentation ability of the engineered strains (**b**) and YZJ015 and YZJ119 which were constructed in our previous studies (**c**). Twenty gram per liter glucose and 80 g/L xylose were used for xylitol production at 42 °C. The values are the means of three biological replicates ± standard deviation (n = 3) at each time point

Constitutive expression of a high activity exogenous xylose reductase, which is the critical enzyme for xylitol production, could facilitate the xylitol accumulation [11]. The *K*cat/*K*m of xylose reductase from *N. crassa* is over 100-fold of xylose reductase from *K. marxianus* [8]. Therefore, *NcXYL1* gene was selected to improve xylitol production in this study. Moreover, overexpression of the xylose-specific transporter *Sc*GAL2N376F could increase the xylose transportation [5, 18]. Then, based on the platform strain, series of strains with improved capacity for xylose–glucose co-utilization were constructed (Fig. 5a). YLM005 produced 18.76 g/L xylitol in 34 h at 42 °C and consumed about 10 g/L xylose before glucose was depleted, confirming its xylose–glucose co-consumption ability (Fig. 5b). Meanwhile, YHY006 and YHY008, corresponding to two and four copies of *NcXYL1* gene expressed in YLM005, produced 32.02, and 40.00 g/L xylitol, respectively, from 80 g/L xylose and 20 g/L glucose (Fig. 5b). With increased copies of *NcXYL1* expressed in *K. marxianus*, the efficiency of xylitol production was improved. Then, xylose-specific transporter *Sc*GAL2N376F was overexpressed in strain YHY008 to further facilitate the co-utilization of glucose and xylose. YHY010 and YHY013, with one and two copies of *ScGAL2N376F* in YHY008, produced 49.14 and 60.05 g/L xylitol in 34 h, with productivity of 1.45 and 1.77 g/(L h), respectively (Fig. 5b). As compared with YHY008, the xylitol production of YHY013 was dramatically increased, indicating that the xylose-specific transporter enhanced the xylitol production during xylose–glucose co-fermentation.

Though overexpression of xylose reductase and/or of the xylose-specific transporter enhanced xylitol production, highly efficient xylitol production in the presence of glucose was not guaranteed with these modifications in other strains. YZJ119 was constructed in our previous study and harbored multiple copies of *NcXYL1* and xylose-specific transporter *Sc*GAL2N376F (as YHY013 does). However, it was not constructed from the glucose derepression platform strain and produced only 26.48 g/L xylitol in 46 h from 20 g/L glucose and 80 g/L xylose (Fig. 5c). This production level was much lower than that of YHY013 (Fig. 5b). The glucose repression in YZJ119 was released only at the sugar transportation stage by overexpression of the xylose-specific transporter, and the endogenous xylose metabolic pathway was still repressed even glucose was used up quickly [5, 18]. As a result, once glucose is exhausted, the supply of energy and coenzyme needed for *Nc*XR to produce xylitol may stop. Thus, large amounts of glucose may be needed for high xylitol production. As to YHY013, xylose assimilation related genes (including the transport stage and metabolic stage) were released from glucose repression, the xylose metabolic pathway was intact, and glucose and xylose supplied the needed energy and coenzyme for xylitol production simultaneously. After glucose was used up, a portion of xylose was metabolized to supply the energy and coenzyme continuously. Therefore, even with lower glucose supply, substantial amounts of xylitol can be produced and additional supplementation of co-substrate was not necessary which simplified the fermentation procedure. The glucose concentration in XML is only a quarter of the xylose and the glucose concentration is even lower in corncob hydrolyaste. As a result, the YZJ119 cannot utilize these biomass for xylitol production as efficiently as YHY013 does. Similarly, YZJ015, carrying the same copies of *NcXYL1* as YHY008 does but did not release from glucose repression, had splendid performance on xylitol production (71.46 g/L) with xylose as the sole carbon source [11]. Nevertheless, the strain produced only 25.59 g/L xylitol in the presence of glucose (Fig. 5c). The xylose assimilation ability of strain YZJ074, which produced over 300 g/L xylitol with glycerol as co-substrate, was also severely decreased with the presence of glucose [10]. Consequently, the excellent xylose–glucose co-utilization ability of YHY013 verified the advantages and potential applications of the platform strain YLM005.

### Effects of various nitrogen sources on xylitol production from xylose–glucose mixture by *K. marxianus*

YHY013 showed excellent co-fermentation ability while 11.80 g/L xylose was left with 20 g/L glucose and 80 g/L xylose as a starting substrate (Fig. 5b). Then, another key factor, the nitrogen source, in the fermentation was optimized.

**Fig. 6:**
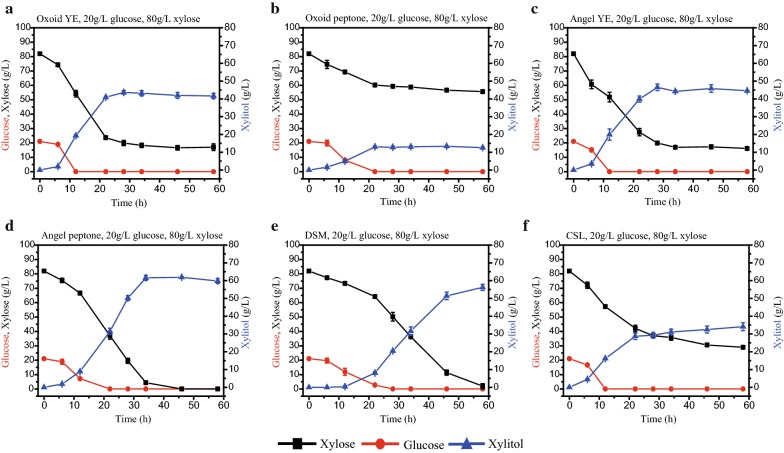
Evaluation of single nitrogen source for xylitol production by YHY013 at 42 °C. The fermentation medium contained 20 g/L glucose and 80 g/L xylose

**Fig. 7:**
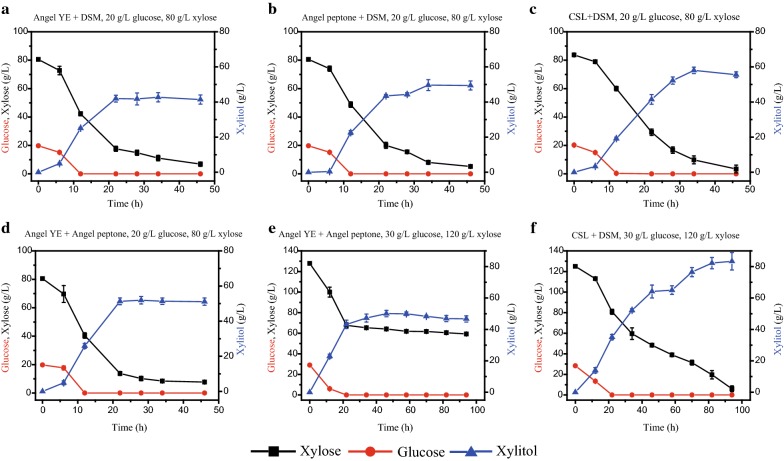
Evaluation of mixed nitrogen sources for xylitol production by YHY013 at 42 °C. The fermentation meidum contained 20 g/L glucose and 80 g/L xylose (**a**–**d**), or 30 g/L glucose and 120 g/L xylose (**e**–**f**)

Here, the efficiency of various nitrogen sources (Oxoid YE, Oxoid peptone, Angel YE, Angel Peptone, CSL, and DSM) for xylitol production was evaluated and presented in Fig. 6. Among these nitrogen sources, the price advantage of CSL and DSM (byproducts of agriculture) makes them very attractive as the nitrogen source for industrial production [38]. With DSM as the nitrogen source, xylitol production (56.22 g/L) was considerable, but xylitol productivity (0.97 g/(L h)) was low, which may be due to its poor performance at the early stage of fermentation (0.36 g/L xylitol was produced in the first 12 h; Fig. 6e). On the contrary, with CSL as the nitrogen source, YHY013 produced considerable amounts of xylitol (16.15 g/L) in the first 12 h, but the final xylitol production (34.07 g/L) and productivity (0.59 g/(L h)) were low (Fig. 6f). YE and peptone from Oxoid Co. are the most expensive materials in this study, but not the best for xylitol production. Most xylose (60.21 g/L) was unused and only 13.03 g/L xylitol was produced with Oxoid peptone (Fig. 6b). With Angel peptone as the nitrogen source, almost all the xylose was consumed, and the xylitol production (61.59 g/L) and productivity (1.81 g/(L h)) were high (Fig. 6d), but Angel peptone is still expensive when compared with CSL and DSM. The efficiency of xylitol production with Angel YE was almost the same as that of Oxoid YE but has a price advantage (produced ~ 45 g/L xylitol in 28 h, Fig. 6a, c). Due to the low xylitol production at the early stage with DSM as the nitrogen source, Angel YE, Angel Peptone, or CSL were mixed with DSM and expected to compensate for the drawback of DSM. As shown in Fig. 7, with DSM mixed with Angel YE, Angel peptone, or CSL as the nitrogen source, strain YHY013 produced 41.90, 49.61, and 58.04 g/L xylitol with productivity of 1.90, 1.46, and 1.70 g/(L h), respectively. The xylitol production with the cheapest combination (DSM and CSL) was the highest. In addition, with the combination of Angel YE and Angel peptone, YHY013 produced 51.28 g/L xylitol from 20 g/L glucose and 80 g/L xylose with productivity of 2.33 g/(L h), which was much faster than combination of CSL and DSM (Fig. 7d). Nonetheless, when higher concentrations of xylose (120 g/L) and glucose (30 g/L) were fermented, the combination of DSM and CSL afforded an obvious advantage over the combination Angel YE + Angel Peptone (Fig. 7e, f). Xylose was consumed thoroughly and 83.39 g/L xylitol was produced by YHY013 with CSL and DSM as the nitrogen source, while 46 g/L xylose was left and only 50.11 g/L xylitol was produced with Angel YE and Angel peptone. The results indicated that the residue of xylose maybe due to the exhaustion of nitrogen source while the slower release of nitrogen source from the combination of CSL and DSM could supply nitrogen for a longer time. Therefore, the combination of CSL and DSM was the best choice for xylitol production in this study and was selected for the subsequent fermentation. It offered obvious price and efficiency advantages especially in medium containing higher concentration of sugars, which is much more suitable for industrial application.

### Xylitol production in a fermenter

**Fig. 8:**
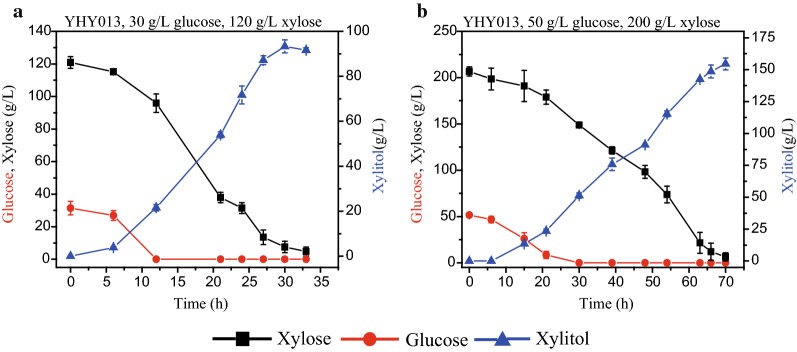
*K. marxianus* YHY013 co-fermentation of glucose and xylose in a fermenter at 42 °C. The fermentation medium contained 30 g/L glucose and 120 g/L xylose (**a**), or 50 g/L glucose and 200 g/L xylose (**b**)

Because the control of oxygen availability is important during xylitol production, a fermenter was used to evaluate the xylitol production. After condition optimization, a two-stage xylitol production strategy (300 rpm with 0.5 vvm was used in the first 12 h, then fermentation was conducted at 350 rpm with 0.5 vvm) was implemented (Additional file 3). Under this condition, YHY013 produced 93.33 g/L xylitol with productivity of 3.11 g/(L h) from 120.89 g/L xylose and 31.4 g/L glucose (Fig. 8a). Furthermore, fermentation with higher sugar concentration was conducted. With 206.63 g/L xylose and 51.52 g/L glucose, 154.50 g/L xylitol was produced with productivity 2.21 g/(L h) (Fig. 8b). These results indicated that YHY013 could produce considerable amounts of xylitol from mixed sugars of glucose and xylose with inexpensive nitrogen source.

**Fig. 9:**
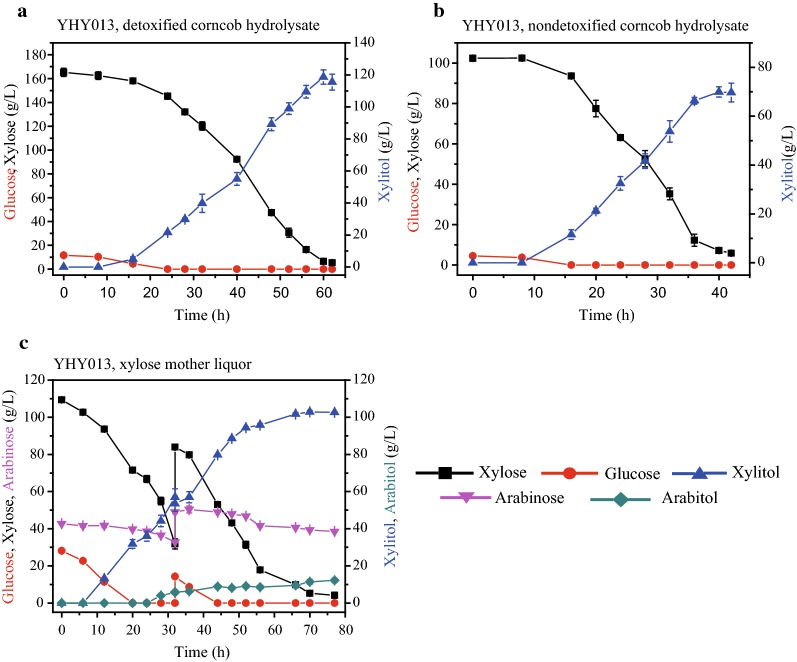
Xylitol production from detoxified corncob hydrolysate, nondetoxified hydrolysate, or XML in fermenter at 42 °C. The concentration of arabinose in the detoxified (**a**) or nondetoxified corncob hydrolysate (**b**) was very low and is not shown. During the fermentation of XML (**c**), 50 mL of non-sterilized XML (containing about 400 g/L of xylose, without diluted) was fed into the fermenter directly when the xylose concentration in the medium decreased to ~ 30 g/L

One of the advantages of bio-production of xylitol is no requirement of pure xylose. In this study, production of xylitol from corncob hydrolysate or XML by *K. marxianus* YHY013 was evaluated. First, both the nondetoxified and detoxified hydrolysates were used to determine the utilization of corncob hydrolysate by *K. marxianus* YHY013. With the concentrated detoxified hydrolysate (containing 118.31 g/L xylose), YHY013 produced 82.85 g/L xylitol with productivity of 2.44 g/(L h) (Additional file 2: Fig. S2). A higher concentration of the detoxified hydrolysate (containing 165.29 g/L xylose) led a higher production of xylitol (118.63 g/L) with productivity of 1.98 g/(L h) at 42 °C (Fig. 9a, Table 3). As far as we know, the xylitol production here was only slightly lower than that in Jiang’s work, which produced 120 g/L xylitol [39] (Table 3). In their study, however, the fermentation was conducted with a much higher initial inoculation at a lower temperature (30 °C) (Table 3). Because the value of xylitol is not very high, preparing cells at such high density for fermentation is not economical, and fermentation at a lower temperature could further increase the cost of temperature control. Moreover, YHY013 can effectively utilize the nondetoxified hydrolysate for xylitol production (Table 3). From the concentrated nondetoxified hydrolysate (containing 102.39 g/L xylose), 69.94 g/L xylitol was produced with productivity of 1.75 g/(L h) (Fig. 9b, Table 3). The xylitol production from nondetoxified diluted-acid pretreatment hydrolysate by YHY013 was attractive when compared with other reports (Table 3).

**Table 3:** Comparison of xylitol production from the detoxified or nondetoxified corncob hydrolysate by yeast

Strains	Temperature (°C)	Initial cell density (g/L)	Xylose (g/L)	Glucose (g/L)	Xylitol production (g/L)	Productivity (g/(L h))	Yield	Reference
*Detoxified*								
*C. tropicalis* W103	35	0.3	65.9	10	45.4	0.71	0.71	[44]
*C. tropicalis*	30	2.0	100	–	75.1	2.01	0.75	[45]
*C. tropicalis*	30	4.0% (v/v)^a^	40.16	6.5	22.63	0.47	0.57	[46]
*C. athensensis* SB18	30	0.5	123.42	22	100.01	0.98	0.81	[47]
*C. tropicalis*	30	1.5	116.7	–	96.5	1.01	0.83	[48]
*C. maltosa*	30	High^b^	150	–	120	2.50	0.81	[39]
*C. guilliermondii* FTI 20037	30	1	74.5	15	55	0.57	0.87	[49]
*C. guilliermondii* FTI 20037	30	1	57.50	7.05	36.11	0.75	0.70	[50]
*C. tropicalis* UFMG BX12-a	30	–^c^	~ 52.78	–	42.17	0.88	0.92	[51]
*S. cerevisiae*	30	50	48.7	59.3	37.9	0.39	0.63	[52]
*K. marxianus* YHY013	42	0.42	165.29	11.67	118.63	1.98	0.75	This study
*Nondetoxified*								
*C. tropicalis* M2012462	30	10% (v/v)^a^	~ 55	–	38.8	0.46	0.7	[53]
*C. tropicalis* JH030	30	1–1.5	45.8	8.7	31.1	0.44	0.71	[54]
*C. tropicalis* W103	35	0.5	53.29	8.69	17.1	0.24	0.32	[55]
*C. tropicalis*	30	–^c^	50.6	7	40	0.42	0.79	[56]
*C. tropicalis*	30	–^c^	~ 57	~ 7	41	0.43	0.73	[57]
*S. cerevisiae*	30	0.42	65	7	22.4	0.844	–	[58]
*C. tropicalis* M2012462	35	1.6	~ 50	~ 6	35.6	0.94	0.71	[59]
*K. marxianus* YHY013	42	0.42	102.39	4.49	69.94	1.75	0.73	This study

^a^ In some reports, the inoculation was presented as the ratio of seed culture volume to fermentation volume

^b^ After incubation for 24 h, all the yeast cells were harvested by centrifugation and resuspended in an isometric substrate solution

^c^ Cells were immobilized for fermentation

XML was also used to evaluate the xylitol-producing ability of YHY013. XML is an organic pollutant and accumulated during xylose extraction from corncobs or sugarcane bagasse. As the very high sugar concentration, XML was diluted for use. Wang et al. [40] showed that high xylitol production (95 g/L xylitol produced in 96 h) from XML was achieved by the cooperation of *Candida tropicalis* and recombinant *Bacillus subtilis* for degradation of inhibitors and metabolism of sugars. In this study, 101.75 g/L xylitol was produced by YHY013 in 66 h at 42 °C (Fig. 9c). The xylitol production was higher and the operation was much easier. Nonetheless, a portion of arabinose was converted into arabitol during this fermentation (Fig. 9c), which was used up in Wang’s work. More work will be focused on this challenging issue in the future.

The corncob hydrolysate, containing large amount of xylose, is good feedstock for bioconversion of xylitol [41]. However, it also contains large amounts of inhibitors, such as furfural and 5-hydroxymethylfurfural (HMF), due to the acid pretreatment of corncobs [42]. The concentrations of furfural and 5-HMF were determined before and after fermentation and the results showed that they were degraded during the fermentation of YHY013 (Additional file 1: Table S3). As reported in *S. cerevisae* and our previous report, the degradation may be also based on the NADPH-dependent reduction [14, 43]. The inhibitor-tolerant ability of *K. marxianus* YHY013 made it more suitable for lignocellulosic biomass utilization [5]. Besides, inexpensive nitrogen source (CSL + DSM) further improved the application of YHY013 for industrial production. Taken together, our study not only presented the great potential of platform strain YLM005 but also advanced the manufacture of value-added products from lignocellulosic biomass.

## Conclusions

The glucose repression on xylose utilization was mainly by regulating the expression of xylitol dehydrogenase gene (*KmXYL2*). The *Km*HXK1 and *Km*SNF1 were both involved in the glucose repression. However, the disruption of *Km*MIG1 or many other candidate targets interacting with SNF1 did not release the glucose repression on xylose. The disruption of the downstream genes of SNF3 or PKA signal pathway also did not release the repression. Therefore, other unknown pathway which different to the glucose repression of xylose utilization in *S. cerevisiea* may exist in *K. marxianus*. The analysis of the glucose repression here contributed to the construction of the repression-released platform strain YLM005. The efficient xylose–glucose co-consumption and xylitol production strain YHY013, constructed from YLM005, verified the advantages and potential applications of the platform strain. With inexpensive CSL and DSM as the nitrogen source, and with detoxified or nondetoxified corncob hydrolysate as the substrate, 118.63 or 69.94 g/L xylitol was produced. Besides, YHY013 effectively utilized XML for xylitol production (101.75 g/L). This study further revealed the glucose repression mechanisms of *K. marxianus*, and the platform strain simplified the engineering approach to obtain robust strains for industrial fermentation with lignocellulosic biomass.

## Additional files

**Additional file 1: Table S1.** Plasmids used in this study. **Table S2.** Primers used in this study. **Table S3.** The concentrations of furfural and 5-HMF before and after fermentation with *K. marxianus* YHY013.

**Additional file 2: Fig. S1.** The growth of various strains on YP plates containing various sugars with or without 0.01% 2-DG at 30 ^o^C. **Fig. S2.** *K. marxianus* YHY013 fermented concentrated detoxified corncob hydrolysate containing 118.31 g/L xylose in fermenter at 42 ^o^C.

**Additional file 3.** Optimization of the oxygen availability for the fermentation.
