# Supplementary material for: Release of glucose repression on xylose utilization in Kluyveromyces marxianus to enhance glucose-xylose co-utilization and xylitol production from corncob hydrolysate
Source: Microb Cell Fact. 2019 Feb 1;18:24. doi: 10.1186/s12934-019-1068-2 (PMC6359873; doi:10.1186/s12934-019-1068-2)
Supplement: Supplementary file 1 — Additional file 1: Table S1. Plasmids used in this study. Table S2. Primers used in this study. Table S3. The concentrations of furfural and 5-HMF before and after fermentation with K. marxianus YHY013. [file 12934_2019_1068_MOESM1_ESM.docx]

**Additional file 1**

**Release of glucose repression on xylose utilization in *Kluyveromyces marxianus* to enhance glucose–xylose co-utilization and xylitol production from corncob hydrolysate**

Yan Hua ^a,b^, Jichao Wang^a^, Yelin Zhu ^a^, Biao Zhang ^a^, Xin Kong ^a,b^, Wenjie Li ^a^, Dongmei Wang ^a,b^, Jiong Hong^a,b,^*

^a^ School of Life Sciences, University of Science and Technology of China, Hefei, Anhui 230027, P. R. China

^b^ Hefei National Laboratory for Physical Science at the Microscale, Hefei, Anhui 230026, PR China

*Correspondence:

Jiong Hong, [hjiong@ustc.edu.cn](mailto:hjiong@ustc.edu.cn), Phone: +86 551-63600705, Fax: +86 551-63601443

**Table S1 Plasmids used in this study**

**Table S2 Primers used in this study**

**Table S3** **The concentrations of furfural and 5-HMF before and after fermentation with *K. marxianus* YHY013**

**Table S1 Plasmids used in this study**

| Plasmids | Description | Reference |
| --- | --- | --- |
| yEUGAP | *Amp^R^, ScURA3*, *P_KmTDH3,_ T_KmTDH3_* | [[1](#_ENREF_1)] |
| pMD18T-ΔScURA3 | *Amp^R^,* Δ*ScURA3* | [[1](#_ENREF_1)] |
| pZJ011 | *ScURA3, P_KmTDH3_-NcXYL1-T_ScTDH3_,* *P_ScTDH3_-NcXYL1-T_ScTDH3_* | [[1](#_ENREF_1)] |
| pZJ012 | *ScLeu2, P_KmTDH3_-NcXYL1-T_ScTDH3_,* *P_ScTDH3_-NcXYL1-T_ScTDH3_* | [[1](#_ENREF_1)] |
| pZJ061 | *ScURA3, P_ScTDH3_-ScGal2-N376F-T_ScTDH3_* | [[2](#_ENREF_2)] |
| pZJ041 | *ScURA3, P_ScTDH3_-KmZwf-T_ScTDH3_* | [[2](#_ENREF_2)] |
| YEGAP-GLK1 | *Amp^R^*, *ScTRP1*, P_KmTDH3_-*KmGLK*-T_KmTDH3_ | [[3](#_ENREF_3)] |
| pKmCYR1 | *Amp^R^, KmCYR1* | This study |
| pKmRAS | *Amp^R^, KmRAS* | This study |
| pKmSNF1 | *Amp^R^, KmSNF1* | This study |
| pKmCAT8 | *Amp^R^, KmCAT8* | This study |
| pKmADR1 | *Amp^R^, KmADR1* | This study |
| pKmNRG1 | *Amp^R^, KmNRG1* | This study |
| pKmMIG1 | *Amp^R^, KmMIG1* | This study |
| pKmMSN2 | *Amp^R^, KmMSN2* | This study |
| pKmRDS2 | *Amp^R^, KmRDS2* | This study |
| pKmRGT1 | *Amp^R^, KmRGT1* | This study |
| pKmCYR1-U | *Amp^R^, KmCYR1* inserted with *ScURA3* | This study |
| pKmRAS-U | *Amp^R^, KmRAS* inserted with *ScURA3* | This study |
| pKmSNF1-U | *Amp^R^, KmSNF1* inserted with *ScURA3* | This study |
| pKmCAT8-U | *Amp^R^, KmCAT8* inserted with *ScURA3* | This study |
| pKmADR1-U | *Amp^R^, KmADR1* inserted with *ScURA3* | This study |
| pKmNRG1-U | *Amp^R^, KmNRG1* inserted with *ScURA3* | This study |
| pKmMIG1-U | *Amp^R^, KmMIG1* inserted with *ScURA3* | This study |
| pKmMSN2-U | *Amp^R^, KmMSN2* inserted with *ScURA3* | This study |
| pKmRDS2-U | *Amp^R^, KmRDS2* inserted with *ScURA3* | This study |
| pKmRGT1-U | *Amp^R^, KmRGT1* inserted with *ScURA3* | This study |

**Table S2 Primers used in this study**

| Primers | Sequences* |
| --- | --- |
| KmCYR1H2F | 5′-GTGCCGTCAAGTTGTCAAGTTTGAG-3′ |
| KmCYR1H2R | 5′-GAACAAAGGAAGCATTTCCGCGCTG-3′ |
| KmRASHF | 5′-GTACTGTAACGACTGCTTGAAGAAGC-3′ |
| KmRASHR | 5′-GGAAAGGAAGGTGTGGTTAAACAGAG-3′ |
| KmSNF1HF | 5′-CTGTCGGAAGAAGAATGGAG-3′ |
| KmSNF1HR | 5′-TAATAACGCGGGGATCAGC-3′ |
| KmMIG1F | 5′-CAAGCCACACCATTCCTTTTTATC-3′ |
| KmMIG1R | 5′-TATCCAAGCTTCTGAGCGAC-3′ |
| KmCAT8HF | 5′-CGACTCGGTACATTCTAGCCATG-3′ |
| KmCAT8HR | 5′-CCTCGTTATGTCTCTCTTTTCCCTG-3′ |
| KmADR1HF | 5′-TCACTCATGCCCAATACGCATCATG-3′ |
| KmADR1HR | 5′-CTTAGCTCTGCAAAGATACTCAACAC-3′ |
| KmNRG1HF | 5′-TATGGCCTATGCTATGGGC-3′ |
| KmNRG1HR | 5′-CTCGTCCCAGAAAGCTTAAG-3′ |
| KmMSN2F | 5′-CTTGTTCATAGAAGTTGTTAA-3′ |
| KmMSN2R | 5′-CTATTTGCTGGATGAGTTTGT-3′ |
| KmRDS2F | 5′-ACGGTTACGTTCCTTCATAA-3′ |
| KmRDS2R | 5′-TATCCAGGAGATCGACCAAT-3′ |
| KmRGT1F | 5′-ATTTTCAGACTTCTCGGAAG-3′ |
| KmRGT1R | 5′- CTATACTAAGTCCTGATCGGC -3′ |
| dKmCAT8F | 5′-TGAACTCAGTGTCTATTTTCTAC-3′ |
| dKmCAT8R | 5′-CATTACAAAAACAAATCCACTAC-3′ |
| dKmNRG1F | 5′-TGATTAGGTTCTACGATTTCTGAG-3′ |
| dKmNRG1R | 5′-CATATGGGAACAAAATAAAGCAATTC-3′ |
| dKmMIG1F | 5′-CCTAGTTTCTAGTTCCCAAT-3′ |
| dKmMIG1R | 5′-CCTTTAAACAACAACACCCC-3′ |
| dKmMSN2F | 5′-CACATTCTTGATCGCAGGC-3′ |
| dKmMSN2R | 5′-ATTCATTTCGCCGCAATTAC-3′ |
| dKmRDS2F | 5′-CCTCTCGACATAAATGCC-3′ |
| dKmRDS2R | 5′-AAAGCTCGAAGCAGGCTT-3′ |
| dKmRGT1F | 5′-GGTTGGAGTTGCTGACTGA-3′ |
| dKmRGT1R | 5′-CTCGTTGCAATCTTGTTCT-3′ |
| SCURA3-SMAI-FULL-F | 5′-TCCCCCGGGTATTTAGAAAAATAAACAAATAG-3′ |
| SCURA3-SMAI-FULL-R | 5′-TCCCCCGGGAATGCGTACTTATATGCGTC-3′ |
| RT-XR-F | 5′-GGTATCGCCGTCATTCCAAAGTC-3′ |
| RT-XR-R | 5′-TGGTCCAAGCCGTTGATCTCC-3′ |
| RT-XDH-F | 5′-GGTAAATCGCCTGAAATCGCTATTG-3′ |
| RT-XDH-R | 5′-ACATCATCTCTACCCATACCCACTTG-3′ |
| RT-XK-F | 5′-CGCAAGGACGCCAAGAATATCG-3′ |
| RT-XK-R | 5′-AGGCACCACCGACGAAGAAC-3′ |
| RT-ACT-F | 5′-CTGTCTGGATTGGTGGTTCTA-3′ |
| RT-ACT-R | 5′-TTCGTCGTATTCTTGCTTTGAG-3′ |

*The restriction enzyme sites are underlined.

**Table S3** **The concentration of furfural and 5-HMF before and after fermentation with *K. marxianus* YHY013**

|  | Xylose  (g/L) | Before fermentation | |  | After fermentation | |
| --- | --- | --- | --- | --- | --- | --- |
|  |  | Furfural (mg/L) | 5-HMF (mg/L) |  | Furfural (mg/L) | 5-HMF (mg/L) |
| Xylose mother Liquor | 109.44±0.13 | 311.53±6.38 | 177.29±3.67 |  | - | - |
| Detoxified hydrolysate | 165.29±3.41 | - | 294.14±5.93 |  | - | - |
| Non-detoxified hydrolysate | 102.39±1.40 | - | 703.01±6.70 |  | - | - |

-, Not be detected.

**References**

1. Zhang, J., Zhang, B., Wang, D. M., Gao, X. L., Hong, J., Xylitol production at high temperature by engineered *Kluyveromyces marxianus*. Bioresour. Technol. 2014;152**:** 192-201.

2. Zhang, B., Zhang, J., Wang, D., Han, R., Ding, R., Gao, X., Sun, L., Hong, J., Simultaneous fermentation of glucose and xylose at elevated temperatures co-produces ethanol and xylitol through overexpression of a xylose-specific transporter in engineered *Kluyveromyces marxianus*. Bioresour. Technol. 2016;216**:** 227-37.

3. Zhang, G., Lu, M., Wang, J., Wang, D., Gao, X., Hong, J., Identification of hexose kinase genes in *Kluyveromyces marxianus* and thermo-tolerant one step producing glucose-free fructose strain construction. Sci. Rep. 2017;7**:** 45104.
