# Supplementary material for: Release of glucose repression on xylose utilization in Kluyveromyces marxianus to enhance glucose-xylose co-utilization and xylitol production from corncob hydrolysate
Source: Microb Cell Fact. 2019 Feb 1;18:24. doi: 10.1186/s12934-019-1068-2 (PMC6359873; doi:10.1186/s12934-019-1068-2)
Supplement: Supplementary file 2 — Additional file 2: Fig. S1. The growth of various strains on YP plates containing various sugars with or without 0.01% 2-DG at 30 oC. Fig. S2. K. marxianus YHY013 fermented concentrated detoxified corncob hydrolysate containing 118.31 g/L xylose in fermenter at 42 oC. [file 12934_2019_1068_MOESM2_ESM.docx]

**Additional file 2**

**Release of glucose repression on xylose utilization in *Kluyveromyces marxianus* to enhance glucose–xylose co-utilization and xylitol production from corncob hydrolysate**

Yan Hua ^a,b^, Jichao Wang^a^, Yelin Zhu ^a^, Biao Zhang ^a^, Xin Kong ^a,b^, Wenjie Li ^a^, Dongmei Wang ^a,b^, Jiong Hong^a,b,^*

^a^ School of Life Sciences, University of Science and Technology of China, Hefei, Anhui 230027, P. R. China

^b^ Hefei National Laboratory for Physical Science at the Microscale, Hefei, Anhui 230026, P. R. China

*Correspondence:

Jiong Hong, [hjiong@ustc.edu.cn](mailto:hjiong@ustc.edu.cn), Phone: +86 551-63600705, Fax: +86 551-63601443

**Fig. S1 The growth of various strains on YP plates containing various sugars with or without 0.01% 2-DG at 30 ^o^C.**

**Fig. S2 *K. marxianus* YHY013 fermented concentrated detoxified corncob hydrolysate containing 118.31 g/L xylose in fermenter at 42^o^C.**


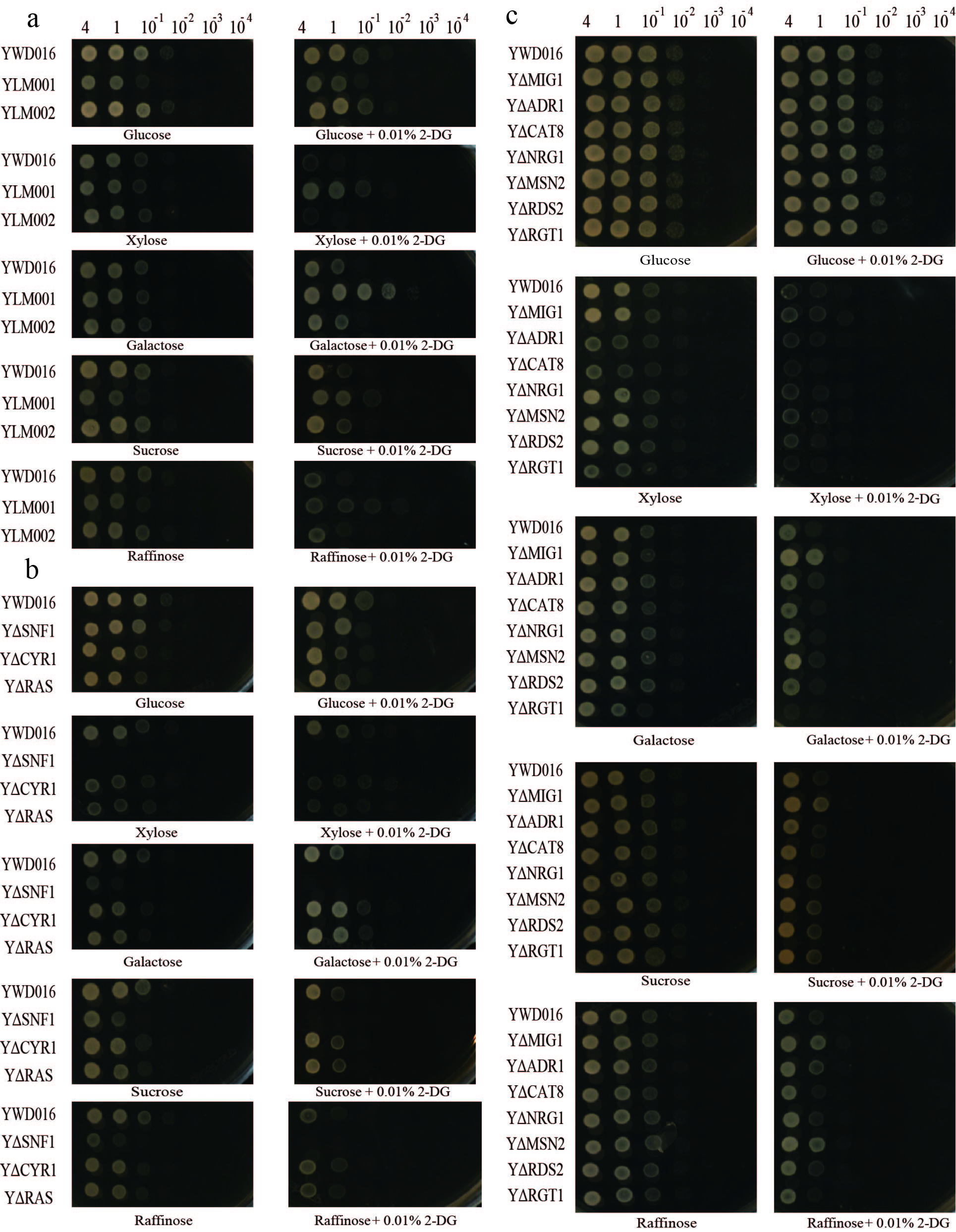


**Fig. S1** The growth of various strains on YP plates containing various sugars with or without 0.01% 2-DG at 30 ^o^C. The dropped cell density are 4, 1, 10^-1^, 10^-2^, 10^-3^, 10^-4^ (OD_600_, left to right). (a) YWD016, YLM001, and YLM002; (b) YWD016, YΔSNF1, YΔCYR1, and YΔRAS; (c) YWD016, YΔMIG1, YΔADR1, YΔCAT8, YΔNRG1, YΔMSN2, YΔRDS2, and YΔRGT1. YWD016 was served as a non-disruption control.


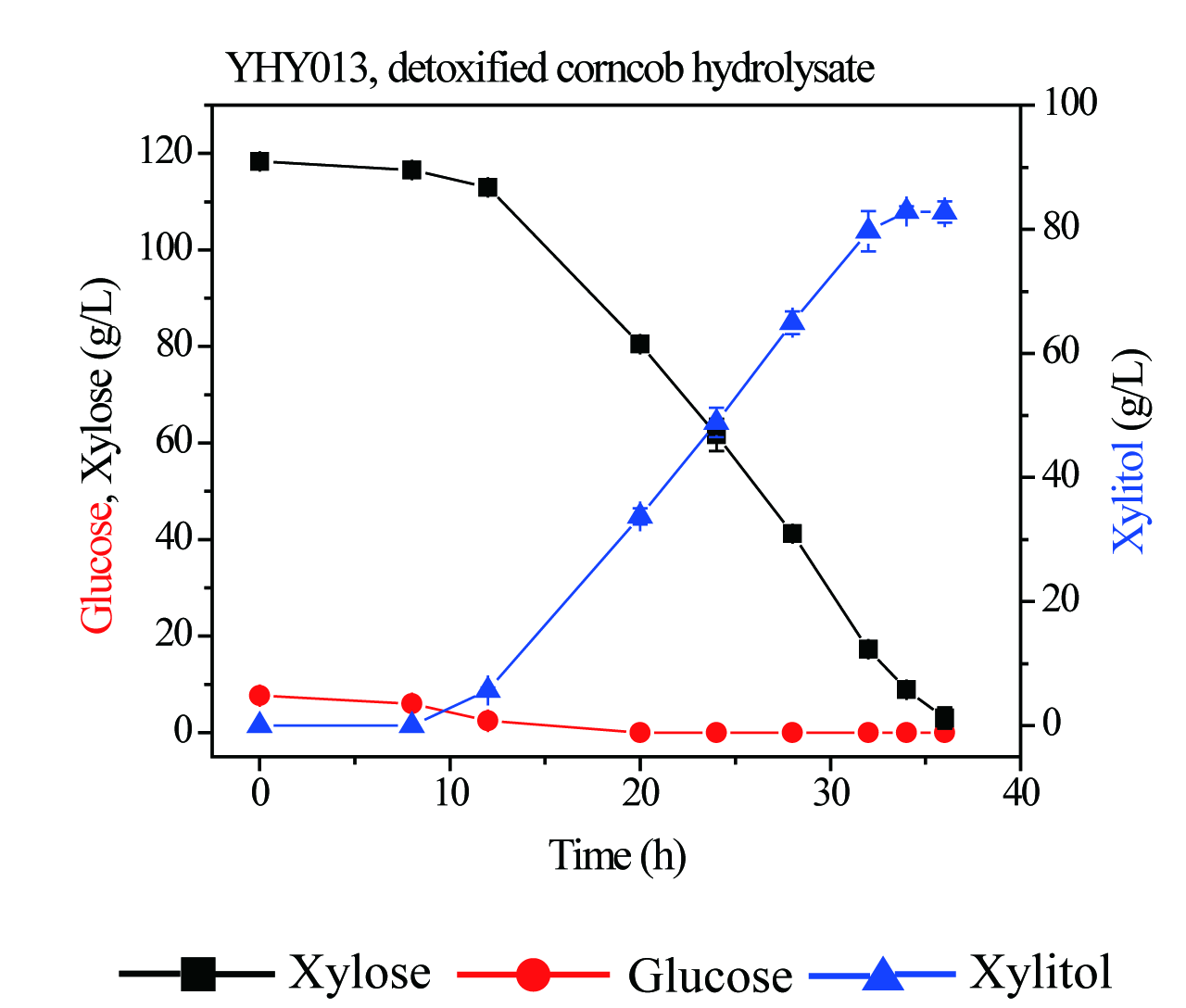


**Fig. S2** *K. marxianus* YHY013 fermented concentrated detoxified corncob hydrolysate containing 118.31 g/L xylose in fermenter at 42^o^C.
