# Supplementary material for: Release of glucose repression on xylose utilization in Kluyveromyces marxianus to enhance glucose-xylose co-utilization and xylitol production from corncob hydrolysate
Source: Microb Cell Fact. 2019 Feb 1;18:24. doi: 10.1186/s12934-019-1068-2 (PMC6359873; doi:10.1186/s12934-019-1068-2)
Supplement: Supplementary file 3 — Additional file 3. Optimization of the oxygen availability for the fermentation. [file 12934_2019_1068_MOESM3_ESM.docx]

**Additional file 3**

**Release of glucose repression on xylose utilization in *Kluyveromyces marxianus* to enhance glucose–xylose co-utilization and xylitol production from corncob hydrolysate**

Yan Hua ^a,b^, Jichao Wang^a^, Yelin Zhu ^a^, Biao Zhang ^a^, Xin Kong ^a,b^, Wenjie Li ^a^, Dongmei Wang ^a,b^, Jiong Hong^a,b,^*

^a^ School of Life Sciences, University of Science and Technology of China, Hefei, Anhui 230027, P. R. China

^b^ Hefei National Laboratory for Physical Science at the Microscale, Hefei, Anhui 230026, PR China

*Correspondence:

Jiong Hong, [hjiong@ustc.edu.cn](mailto:hjiong@ustc.edu.cn), Phone: +86 551-63600705, Fax: +86 551-63601443

**Optimization of the oxygen availability for the fermentation**

As oxygen availability is important for fermentation, it was optimized with various agitation and aeration rates by using a fermenter to improve the xylitol production (Table S3). The highest productivity (2.5 g/[h⋅L]) was obtained with agitation at 350 rpm and aeration of 0.5 vvm (volume of air per volume of the medium per minute). However, only a small amount of xylitol (~5 g/L) was accumulated in the first 12 h at 350 or 400 rpm agitation speed. The agitation speed of 300 rpm at an aeration rate of 0.5 vvm or 1 vvm resulted in a higher xylitol production (~23 g/L) in the first 12 h (Table S1). These results indicated that agitation of 300 rpm was better for xylitol production in the first 12 h. Nonetheless, at this lower agitation speed, the xylose consumption decreased at the later stage with 49.86 and 39.57 g/L xylose was still present at 33 h, respectively, while xylose was almost fully consumed at 350 or 400 rpm within the same timeframe. Because the xylose metabolic pathway of YHY013 is intact, xylitol accumulation is mostly due to a redox imbalance in the cytoplasm. Excessive oxygen led to oxidation of NADH or NADPH to NAD^+^ or NADP^+^, which favor xylitol to xylulose and as a result less xylitol was accumulated [[1](#_ENREF_1)]. That may explain why a lower agitation speed (300 rpm) led to a higher xylitol production in the first 12 h. Nevertheless, a certain amount of oxygen is necessary to maintain the supply of the coenzyme and energy for cell growth and fermentation. Thus, at the later stage with higher OD_600_ of strain, xylose could not be further consumed with lower agitation speed (300 rpm). Therefore, a two stage xylitol production strategy (300 rpm with 0.5 vvm was used here in the first 12 h, then fermentation was conducted at 350 rpm with 0.5 vvm) was implemented and YHY013 produced 93.33 g/L xylitol with productivity of 3.11 g/(L⋅h).

**Table S1** **The** **effect of oxygen availability in a fermenter on xylitol production***

| Agitation (RPM) | Aeration rate (vvm) | Xylitol production in 12 h (g/L) | Final xylitol production (g/L) | Xylose residue  (g/L) | Fermentation time (h) |
| --- | --- | --- | --- | --- | --- |
| 300 | 0.5 | 22.30±1.18 | 59.83±3.06 | 49.86±2.07 | 33 |
| 300 | 1 | 23.77±2.70 | 68.52±4.40 | 37.57±4.41 | 33 |
| 350 | 0.5 | 5.42±0.55 | 83.28±6.85 | 3.42±0.84 | 33 |
| 350 | 1 | 5.03±0.76 | 76.75±2.73 | 4.61±0.69 | 33 |
| 400 | 0.5 | 4.86±0.92 | 76.90±2.13 | 4.79±2.23 | 33 |
| 400 | 1 | 4.62±0.56 | 73.72±1.81 | 4.62±0.56 | 33 |

* The fermentation medium contained 10 g/L CSL, 10 g/L DSM, 30 g/L glucose, and 120 g/L xylose.

**References**

1. Pal, S., Choudhary, V., Kumar, A., Biswas, D., Mondal, A. K., Sahoo, D. K., Studies on xylitol production by metabolic pathway engineered *Debaryomyces hansenii*. Bioresour. Technol. 2013;147**:** 449-55.
